# Comparison of different feeding regimes after pancreatoduodenectomy - a retrospective cohort analysis

**DOI:** 10.1186/s12937-017-0265-2

**Published:** 2017-07-04

**Authors:** Théophile Guilbaud, David Jérémie Birnbaum, Sandrine Loubière, Julien Bonnet, Sophie Chopinet, Emilie Grégoire, Stéphane Berdah, Jean Hardwigsen, Vincent Moutardier

**Affiliations:** 1 Department of Digestive Surgery, Hôpital Nord, Aix-Marseille University, Chemin des Bourrely 13915, cedex 20 Marseille, France; 2 Self perceived Health Assessment Research Unit and Department of Public health, Aix-Marseille University, 27 Boulevard Jean Moulin, 13005, cedex 20 Marseille, France; 3 Department of Digestive Surgery and Liver Transplantation, Hôpital La Timone, Aix-Marseille University, 264 Rue Saint-Pierre 13385, cedex 20 Marseille, France

**Keywords:** Pancreaticoduodenectomy, Gastrojejunostomy tube, Enteral nutrition, Postoperative pancreatic fistula, Delayed gastric empty, Cost savings

## Abstract

**Background:**

Delayed gastric emptying (DGE) is the most frequent pancreatic specific complication (PSC) after pancreaticoduodenectomy (PD). Several gastric decompression systems exist to manage DGE. Patients with a pancreatic tumor require prolonged nutrition; however, controversies exist concerning nutrition protocol after PD. The aim of the study was to assess the safety and efficacy of nasogastric (NG), gastrostomy (GT), and gastrojejunostomy (GJ) tubes with different feeding systems on postoperative courses.

**Methods:**

Between January 2013 and March 2016, 86 patients underwent PD with pancreaticogastrostomy. Patients were divided into three groups: GJ group with enteral nutrition (EN, *n* = 12, 14%), NG (*n* = 31, 36%) and GT groups (*n* = 43, 50%), both with total parenteral nutrition (TPN).

**Results:**

Patients in the GJ (*n* = 9, 75%) and GT (*n* = 18, 42%) groups had an American Society of Anesthesiologists (ASA) score of 3 more often than those in the NG group (*n* = 5, 16%, *p* ≤ 0.01). Multivariate analysis identified the GT tube with TPN as an independent risk factor of severe morbidity (*p* = 0.02) and DGE (*p* < 0.01). An ASA score of 3, jaundice, common pancreatic duct size ≤3 mm and soft pancreatic gland texture (*p* < 0.05) were found as independent risk factors of PSCs. Use of a GJ tube with EN, GT tube with TPN, jaundice, and PSCs were identified as independent risk factors for greater postoperative length of hospital stay (*p* < 0.01). Mean global hospitalization cost did not differ between groups.

**Conclusion:**

GT tube insertion with TPN was associated with increased severe postoperative morbidity and DGE and should not be recommended. EN through a GJ tube after PD is feasible but does not have clear advantages on postoperative courses compared to an NG tube.

## Background

Pancreatoduodenectomy (PD) is the only available curative treatment for both potentially malignant and malignant neoplasm of the pancreatic head [1, 2]. Even if postoperative mortality after pancreatic resection has been significantly reduced to a rate below 5% in most series [3–6], the morbidity remains high [7–10]. Recently, the concepts of “fast-track” surgery, which are to provide optimal perioperative care, have been proven to significantly reduce complication rates and decrease the length of hospital stay [11–13].

Delayed gastric emptying (DGE) is the most frequent complication after PD (15%–60%) [14–18]. This complication is rarely life threatening but increases significantly the length of hospital stay and the cost and can also impair quality of life [5, 19–22]. Different gastric decompression systems are used in postoperative pancreatic resection: the nasogastric tube (NG tube), gastrostomy tube (GT tube), and gastrojejunostomy tube (GJ tube).

NG tube insertion has been associated with higher inconvenience for patients. In addition, it may cause patient discomfort and be associated with nasal trauma, gastroenteral reflux and respiratory complications and can lead to the dislodgement of the NG tube [23–26]. GT tube insertion has advantages over the NG tube, particularly if prolonged gastric decompression is required, as is often the case after PD, without the discomfort and complications associated with prolonged nasal intubation. To our knowledge, only one randomized study [27] has reported on the GJ tube. Prolonged gastric decompression can be effected through the gastric port if necessary without the discomfort and complications associated with prolonged nasal intubation, and enteral nutrition (EN) may proceed through the jejunal port. This published study showed that insertion of a GJ tube during PD is safe and feasible and may reduce average length of hospital stay. This intervention is also cost effective [27].

Furthermore, patients with a pancreatic tumor are often malnourished and require prolonged nutrition. Different feeding systems have been used after pancreatic resection but controversies exist concerning nutrition [28, 29]. According to the European and American Society for Parenteral and Enteral Nutrition guidelines, for patients requiring prolonged nutritional maintenance, the enteral route has many proven benefits over total parenteral nutrition (TPN): improvement of immune function, preservation of gastrointestinal mucosal integrity, decreased risk of infectious complication, and avoidance of infectious risks associated with a central venous catheter [30]. Furthermore, TPN is associated with an increased number of infectious complications and cost [31, 32]. Both enteral and parenteral feeding is associated with specific complications. There is a lack of specific evidence concerning the optimal feeding strategy after PD. Over the last decade, systematic reviews did not find major differences in outcome between an oral diet, EN via a nasojejunostomy tube, a GJ tube or a jejunostomy tube, and TPN after PD. However, the quality of the included studies was moderate [33, 34].

The primary end point of our study was to compare different gastric decompression systems and feeding routes with an NG tube with TPN, a GT tube with TPN, and a GJ tube with EN after PD with pancreaticogastrostomy in terms of postoperative mortality, overall morbidity and pancreatic specific complications (PSCs).

## Methods

### Data collection

From January 1st, 2013 to March 1st, 2016, patients who underwent PD with pancreaticogastrostomy for pancreatic tumor at Hôpital Nord and Hôpital la Timone (Marseille, France) were included in the study. All patient data were obtained from a prospective database with an additional retrospective medical record review. All surgical indications were discussed by a multidisciplinary pancreatic tumor board consisting of surgeons, radiologists, pathologists, oncologists, and gastroenterologists. Patients with a non resectable pancreatic tumor and palliative surgery were excluded. Patients who underwent PD with pancreaticojejunostomy were excluded to rule out potential bias. Pancreatic lesions were classified according to the World Health Organization classification of the exocrine and endocrine neoplasms of the pancreas [35]. This study examined the following variables: demographic and surgical data (gland texture, operative time, and preoperative blood loss), post-operative morbidity, mortality, and length of hospital stay.

### Surgical procedures

To achieve PD, either the duodenum (pylorus-preserving PD, PPPD) in one center or the antrum (pylorus-resecting PD, PRPD) in the other, was transected, followed by transection of the pancreatic neck, uncinate process, and jejunum distal to the ligament of Treitz. According to center preference, reconstruction was undertaken with a pancreaticogastrostomy, followed by an end-to-side choledochojejunostomy and either antecolic or retrocolic end-to-side duodenojejunostomy in patient with a PPPD or a gastrojejunostomy in patient with a PRPD. Intra-operatively, all resections included regional lymph node dissection. A drain was placed close to the pancreaticgastrostomy and posterior to the choledochojejunostomy. At the end of the procedure, three different gastric decompression and feeding systems were used: a nasal NG tube with TPN (NG group) was inserted in patients with PRPD, and a GT tube plus TPN (GT group) or a GJ tube (MIC GJ-feeding tube, Halyard Health Inc., Alpharetta, GA, USA, GJ group) placement with EN was inserted in patients with PPPD. GT tube or GJ tube placement was followed by stomach fixation to the anterior abdominal wall.

The allocation of gastric decompression device was not randomized. GT tube was used between January 2013 and July 2015, and GJ tube was used from July 2015 to March 2016.

### Nutritional protocol

Preoperatively, patients received immunonutrition for 7 days (Oral Impact). In the NG group and GT group, all patients had TPN (Smofkabiven, Fresenius kabi, Olimel N7 E, Baxter). In the GJ group, EN (Peptamen HN, Nestlé) started 24 h after feeding tube placement, beginning at 500 ml/day on postoperative day (POD) 1. The feeding rate was increased to 1000 ml/day on POD 2 and to 1500 ml/day from POD 3. The gastric port serving for gastric decompression was routinely clamped at POD 7 according to the decision of the surgeon. Patients were then allowed to start oral intake. TPN and EN were discontinued when oral intake was sufficient.

### Postoperative courses and follow-up

Postoperative mortality included all deaths occurring prior to hospital discharge or within 30 days of the surgery.

Postoperative morbidity included all complications following the surgery until discharge and/or readmission within 30 days and was graded based on the Clavien-Dindo classification system. Severe morbidity included all complications graded Clavien-Dindo 3/4. PSCs included postoperative pancreatic fistula (POPF), bile leak, postoperative hemorrhage, intra-abdominal abscess, and DGE. POPF was classified according to the International Study Group of Pancreatic Surgery (ISGPS) [36]. Bile leak was defined as a bilious drainage from drains or bile collection requiring drainage and postoperative bleeding as the requirement of transfusion or endoscopic or operative intervention. DGE was defined and classified based on the ISGPS [37]. Blood examinations and drain amylase/lipase fluid were conducted on POD 1, 3, 5, and 7. Octreotide was given to patients when POPF grades B/C were diagnosed. An oral diet was started on POD 1. Abdominal CT scans were done at the discretion of each surgeon (fevers, elevated white blood count, unexplained hemodynamic instability, gastric empting, bleeding, and abdominal pain). Only DGE grades B and C was evaluated because all patients had a gastric decompression and as a consequence, all patients fulfill the grade A criteria. Duration of gastric decompression through the NG tube, GT tube, GJ tube, and oral intake were at the discretion of the surgical team. After the stomach was kept in charge, GJ tube and GT tube were left in place two or three more days until tolerated and sufficient oral intake and then they were removed*.* All patients were discharged home without GJ tube or GT tube. The drain was removed depending on clinical factors and on the amylase concentration in the drain fluid.

Follow-up examinations were conducted a first time 1 month after discharge then every 6 months for the first 2 years and annually thereafter with clinical examination, biochemical and computed tomography (CT) scans. The patient follow-up was completed in September 2016.

### Cost

The healthcare costs included were those likely to differ across the intervention and control groups during the follow-up period. These costs were those associated with the following: length of hospital stay, duration of EN or TPN, intensive care unit length of stay, and the length of stay after 30-day readmission. All resources consumed were individually and prospectively collected for each patient enrolled in the study. Unit costs for health service use were obtained from the National health insurance system and hospital stay costs were derived from the adjusted diagnosis-related groups (DRGs) costing method using data from the ENCC (2013) [38]. One-day stays were estimated based on the average cost of DRG and the average cost of an intensive care unit stay. For inpatient care during surgery, we used the specific DRG “interventions for liver, pancreas or malignant tumors”; for inpatient care secondary to surgery and readmission, we used the specific DRG “Malignancy of hepatobiliary system or pancreas”. Daily costs for enteral and parenteral nutrition were obtained from the register of the pharmacy service.

### Statistical analysis

The analysis used descriptive statistics without an “a priori” hypothesis. Data are presented as frequencies and percentages for categorical data and median ± standard deviation (SD) for continuous data. Significant differences between groups in demographic characteristics, diagnoses, morbidity, and mortality were examined using a chi-square test or Fisher’s exact test for categorical data and Student’s *t*-test for continuous data, as appropriate. All *p* values were two-tailed and *p* values <0.05 were considered as significant. The full population (including all subjects in the three groups) were used in the primary analysis. Variables meeting a threshold of *p* < 0.20 were included in the multivariate analysis. Kaplan-Meier methods along with the log rank and Wilcoxon tests were used to establish statistical differences in length of hospital stay between the two groups. Length of hospital stay was calculated as the number of days from surgery to the date of discharge. Cox regressions were used to assess the influence of covariates on the length of stay, accounting for attrition issues (death in this case) and potential collinearity. For PSCs or gastroparesis rates, we used generalized linear models (GLM) with appropriate statistical distributions to estimate odds ratios (OR) and 95% confidence intervals (CI) for association between gastric decompression and feeding systems and each dependent variable in full and matched patients.

In a secondary analysis, we used the propensity score matching approach [39] to allow comparison of the NG group and the GJ tube group according to costs with the same baseline characteristics (including age, gender, weight, ASA score, and pancreatic gland texture, *n* = 12 in each group). Given the skewness of the cost data, a GLM with a negative binomial distribution and log link was specified to explore the effects of categorical and continuous variables on variation in costs. SPSS version 20.0, and R statistical software were used for statistical analyses.

## Results

### Patient characteristics

A total of 86 patients (41 men, 45 women) underwent PD with pancreaticogastrostomy in the study period with a median age of 65 years (25 to 80 years). Patients were divided into three groups: GJ group with EN (*n* = 12, 14%, GJ group), NG group with TPN (*n* = 31, 36%, NG group) and GT group with TPN (*n* = 43, 50%, GT group). Demographic data are summarized in Table 1.

**Table 1:** Patient characteristics. Univariate analyses

	Overall	GJ group	NG group	GT group	*p* value		
	(*n* = 86)	(*n* = 12)	(*n* = 31)	(*n* = 43)	GJ vs NG	GJ vs GT	NG vs GT
Age (year), mean (±SD)	65.1 (±10.2)	65.8 (±9.2)	64.9 (±11.9)	63.3 (±10.0)	0.67	0.40	0.70
Female	45 (52)	7 (58)	13 (42)	25 (58)	1.00	0.50	0.24
Active smoker	26 (30)	6 (50)	5 (16)	15 (35)	0.05	0.50	0.11
Jaundice	53 (62)	5 (42)	21 (68)	27 (63)	0.17	0.21	0.81
Biliary stenting	30 (35)	5 (42)	7 (23)	18 (42)	0.27	1.00	0.13
Neoadjuvant therapy	4 (5)	1 (8)	1 (3)	2 (5)	0.49	0.53	1.00
BMI (kg/m^2^), mean (±SD)	24.4 (±4,4)	23.5 (±2)	23.9 (±4,8)	25.2 (±4.7)	0.67	0.13	0.31
Albumin (g/L), mean (±SD)^a^	38.6 (±5.5)	37.7 (±7.0)	39.6 (±5.9)	37.9 (±4.6)	0.41	0.90	0.23
ASA score							
1 or 2	54 (63)	3 (25)	26 (84)	25 (58)			
3	32 (37)	9 (75)	5 (16)	18 (42)	<0.01	0.06	0.02

Values in parentheses are per cent or SD. *BMI* Body Mass Index, *ASA* American Society of Anaesthesiologists status

^a^Preoperative serum albumin level

Briefly, when comparing mean age, gender, active smoker, BMI and preoperative serum albumin level, between the three groups, no differences were observed. Furthermore, the rate of preoperative jaundice, biliary stenting and neoadjuvant therapy were not different between the three groups.

However, patients in the GJ (*n* = 9, 75%) and GT (*n* = 18, 42%) groups had an American Society of Anesthesiologists (ASA) score of 3 more often than those in the NG group (*n* = 5, 16%, GJ group vs NG group, *p* < 0.01, GT group vs NG group, *p* = 0.02).

### Pathological findings

The indications for PD were as follows: 58 pancreatic adenocarcinoma (67%), 10 bile duct carcinoma (12%), 8 intraductal papillary and mucinous neoplasm (9%), 3 pancreatic endocrine tumor (3%), and 7 other malignancies (8%) including ampullary adenocarcinoma, duodenal adenocarcinoma and metastatic pancreatic carcinoma. No difference was observed concerning the final histological diagnosis between the three groups, which are summarized in Table 2.

**Table 2:** Pathological findings. Univariate analyses

	Overall	GJ group	NG group	GT group	*p* value		
	(*n* = 86)	(*n* = 12)	(*n* = 31)	(*n* = 43)	GJ vs NG	GJ vs GT	NG vs GT
PDAC	58 (67)	8 (67)	21 (68)	29 (67)	1,00	1,00	1,00
IPMN	8 (9)	1 (8)	1 (3)	6 (14)	0,49	1,00	0,23
Neuroendocrine tumor	3 (3)	0 (0)	1 (3)	2 (5)	1,00	1,00	1,00
Bile duct carcinoma	10 (12)	2 (2)	3 (10)	5 (12)	0,61	0,64	1,00
Other malignancies	7 (8)	1 (8)	5 (16)	1 (2)	0,49	1,00	0,39

Values in parentheses are per cent. *PDAC* pancreatic ductal adenocarcinoma, *IPMN* intraductal papillary and mucinous neoplasm

### Surgical procedures

All procedures followed laparotomy. PPPD was performed in 55 patients (64%) and PRPD in 31 patients (36%). Eight patients (9%) required portal vein resection (PVR). Reconstruction of the portal vein was performed by simple suture or primary end-to-end anastomosis. Three patients developed postoperative portal vein thrombosis, one in the NG group and two in the GT group. Seven patients (8%) underwent enlarged “en-bloc” resection of adjacent organs (liver resection, *n* = 2; right colon, *n* = 4; transversal colon, *n* = 1).

No differences were observed regarding the pancreatic gland texture, common pancreatic duct size, and pancreatic stenting between the three groups. Overall, median estimated blood loss was 510 ± 365 mL without differences between each group. Perioperative transfusion requirements were similar in the three groups. No intraoperative difficulties related to GT or GJ tube insertion were reported.

Mean operative time for the entire cohort was 342 +/− 115 min and was lower in the NG group (267 +/− 77 min) than in the GJ (454 +/− 128 min) or GT group (392 +/− 91 min, NG group vs GJ and GT groups, *p* < 0.01). Pancreatic surgical procedures are summarized in Table 3.

**Table 3:** Perioperative data. Univariate analyses

	Overall	GJ group	NG group	GT group	*p* value		
	(*n* = 86)	(*n* = 12)	(*n* = 31)	(*n* = 43)	GJ vs NG	GJ vs GT	NG vs GT
PPPD	55 (64)	12	0	43			
PRPD	31 (36)	0	31	0			
Operative time (min), mean (±SD)	342 (±115)	454 (±128)	267 (±77)	392 (±91)	<0,01	1,00	<0,01
Venous resection	8 (9)	1 (8)	4 (13)	3 (7)	1,00	1,00	0,44
Extended resection	7 (8)	0 (0)	4 (13)	3 (7)	0,56	1,00	0,44
Soft pancreatic gland texture	56 (65)	6 (50)	22 (71)	28 (65)	0,29	0,50	0,63
MPD size ≤3 mm	49 (57)	6 (50)	22 (71)	21 (49)	0,29	1,00	0,09
Pancreatic stenting	60 (70)	11 (96)	22 (71)	27 (63)	0,24	0,08	0,62
Perioperative transfusion	18 (21)	0 (0)	10 (32)	8 (19)	0,15	0,17	1,00

Values in parentheses are per cent or SD. *PPPD* pylorus preserving pancreaticoduodenectomy, *PRPD* pylorus resecting pancreaticoduodenectomy, *MPD* main pancreatic duct

### Postoperative mortality and overall morbidity

The mortality and overall morbidity rates were 6% (*n* = 5) and 73% (*n* = 63), respectively, including severe complications (Clavien-Dindo grade 3/4) in 55% of patients (*n* = 47, Table 4). Mortality rate did not differ between the three groups. Five deaths occurred within 30 days of resection: 2 patients (17%) died in the GJ group and 3 patients (7%) in the GT group. All deaths were secondary to multi-visceral and cardiac failures in patients who experienced grade C POPF and postoperative hemorrhage.

**Table 4:** Postoperative mortality, morbidity and PSCs. Univariates analyses

	Overall	GJ group	NG group	GT group	*p* value		
	(*n* = 86)	(*n* = 12)	(*n* = 31)	(*n* = 43)	GJ vs NG	GJ vs GT	NG vs GT
Mortality	5 (6)	2 (17)	0 (0)	3 (7)	0,07	0,30	0,26
Overall morbidity	63 (73)	11 (92)	19 (61)	33 (77)	0,07	0,42	0,20
Severe morbidity	47 (55)	6 (50)	15 (48)	26 (60)	1,00	0,53	0,35
PSCs	47 (55)	7 (58)	15 (48)	25 (58)	0,74	1,00	0,48
Pancreatic fistula	33 (38)	6 (50)	10 (32)	17 (40)	0,31	0,53	0,63
A	16 (19)	2 (17)	6 (19)	8 (19)	1,00	1,00	1,00
B	7 (8)	1 (8)	4 (13)	2 (5)	1,00	0,53	0,23
C	10 (12)	3 (25)	0 (0)	7 (16)	0,02	0,67	0,04
Biliary fistula	9 (10)	0 (0)	1 (3)	8 (19)	1,00	0,17	0,07
Digestive fistula	4 (5)	1 (8)	3 (10)	0 (0)	0,61	0,22	0,07
Haemorrhage	16 (19)	3 (25)	4 (13)	9 (21)	0,38	0,71	0,54
DGE							
No or A	50 (8)	6 (50)	25 (81)	19 (44)			
B or C	36 (42)	6 (50)	6 (19)	24 (56)	0,06	0,75	<0,01
Gastric decompression time (days), mean (±SD)	11 (±10)	17 (±12)	6 (±5)	16 (±13)	<0,01	1,00	<0,01

Values in parentheses are per cent or SD. *PSCs* Pancreatic Specific Complications, *DGE* Delayed gastric empty

The most common catheter-related complication in the GJ group was bowel blockage in 1 (8%) patient, followed by pain and parietal complication (abscess) after removal of gastrostomy in 4 (9%) patients in the GT group, whereas in the NG group in 4 (12.5%) patients, it was displacement. There were no catheter-related mortalities and no reoperation for a catheter- or feeding-related problem, even after removal. Multivariate analysis, including the GJ tube with EN, NG tube with TPN, GT tube with TPN, age, gender, ASA score, BMI, and jaundice, identified the GT tube with TPN as an independent risk factor of severe morbidity (Clavien-Dindo grade 3/4, *p* = 0.02).

### Postoperative pancreatic-specific complications (PSCs)

The overall proportion of PSCs was 55% (*n* = 47, Table 4), and no differences were observed between the three groups. Multivariate analysis, included the GJ tube with EN, NG tube with TPN, GT tube with TPN, age, gender, ASA score, jaundice, common pancreatic duct size ≤3 mm, and pancreatic gland texture. Four independent risk factors were identified for PSCs: an ASA score of 3, jaundice, common pancreatic duct size ≤3 mm and soft pancreatic gland texture (all *p* values were <0.05).

Postoperative DGE (grades B and C) occurred in 36 patients (42%) and was higher in the GT group than in the NG group (GT group vs NG group, *p* < 0.01), but no difference was found between GJ and NG groups. Any patients get off protocol, therefore no patients needed nasogastric tube insertion or TPN in the GJ group. However, gastric decompression mean duration was lower in the NG group (6 days +/− 5) than in the GJ (17 days +/− 12) and GT group (17 days +/− 13, NG group vs GJ group and GT group, *p* < 0.01). In multivariate analysis, including the GJ tube with TPN, NG tube with TPN, GT tube with EN, age, gender, ASA score, active smoker, and jaundice, only GT with TPN was identified as an independent risk factor of DGE (*p* < 0.01).

Based on the ISGPF criteria, 53 of the 86 patients (62%) did not develop a POPF, and 16 patients (19%) developed only biochemical leak (POPF grade A), while a clinically relevant POPF (grade B/C) developed in 17 patients (20%). Grade C POPF was more frequent in the GJ and GT groups than in the NG group (GJ group vs NG group, *p* = 0.02, GT group vs NG group, *p* = 0.04). Four patients required drainage of a postoperative collection in the resection bed. Multivariate analysis including the same factors, identified soft pancreatic gland texture as the only independent risk factor of POPF (*p* = 0.03). It did not highlight GJ tube, GT or feeding strategy as independent risk factors of clinically relevant POPF. Postoperative hemorrhage occurred in 19% (*n* = 16) of the patients. Nine patients had a radiologic embolization and 7 patients required reoperation.

### Length of stay in hospital

The median length of hospital stay was 26 days (+/− 14) and was longer in the GT group than in the NG group (29 days +/− 16 vs 22 days +/− 13, *p* = 0.04). However, no difference was noted between the GJ (27 days +/− 12) and NG groups (*p* = 0.16). Requirement and length of stay in a critical care environment and readmission after 30 days were similar in each group (Table 5).

**Table 5:** Postoperative managements and cost analysis. Univariate analyses

	Overall	GJ group	NG group	GT group	*p* value		
	(*n* = 86)	(*n* = 12)	(*n* = 31)	(*n* = 43)	GJ vs NG	GJ vs GT	NG vs GT
Drainage^a^	4 (5)	1 (8)	1 (3)	2 (5)	0,49	0,53	1,00
Embolization^b^	10 (12)	2 (17)	2 (6)	6 (14)	0,45	1,00	0,57
Reoperation	7 (8)	2 (17)	0 (0)	5 (12)	0,07	0,64	0,07
ICU hospitalization	49 (57)	6 (50)	14 (45)	29 (67)	1,00	0,32	0,06
ICU length of stay (days), mean (±SD)	7 (±7)	5 (±10)	2 (±3)	3 (±8)	0,11	0,78	0,14
Nutrition time (days), mean (±SD)	14 (±14)	13 (±10)	9 (±12)	19 (±16)	0,16	0,96	<0,01
Length of hospital stay (days), mean (±SD)	26 (±14)	27 (±12)	22 (±13)	29 (±16)	0,16	0,81	0,04
30 days readmission	15 (17)	2 (17)	5 (16)	8 (19)	1,00	1,00	1,00
Nutrition cost (euros), mean (±SD)		843 (±336)	773 (±177)	1884 (±252)	1,00	<0,01	<0,01
Global hospitalization cost (euros), mean (±SD)		18,854 (±3127)	14,226 (±1797)	21,026 (±1889)	0,22	1,00	0,01

Values in parentheses are per cent or SD. *ICU* intensive care unit

^a^Percutaneous or endoscopic drainage of a postoperative abdominal collection

^b^Radiological embolization of a postoperative haemorrhage

Multivariate analysis, including the GJ tube with EN, NG tube with TPN, GT tube with TPN, age, gender, ASA score, jaundice, and PSCs, identified four independent risk factors for greater postoperative length of stay: GJ tube with EN, GT tube with TPN, jaundice, and PSCs (all *p* values were <0.01).

### Cost analysis

Nutrition and global hospitalization cost were compared between the three groups (Table 5). The mean duration of nutrition was lower in the NG group (9 days +/− 12) than in the GT group (19 days +/− 16, *p* < 0.01). However, no difference was found between the NG and GJ groups (13 days +/− 10, *p* = 0.16). Mean nutrition cost was similar between the GJ and NG groups (843 euros +/− 336 vs 773 euros +/− 177, *p* = 1.00) and was higher in the GT group (1884 euros +/− 252, *p* < 0.01 vs GJ and NG groups).

According to mean global hospitalization cost, no difference was found between the GJ and NG groups (18,854 euros +/− 3127 vs 14,226 euros +/− 1797, *p* = 0.22). In a secondary analysis, a propensity score matching approach was used to compare the NG and GJ groups with the same baseline characteristics (including age, gender, weight, ASA score, and pancreatic gland texture, *n* = 12 in each group). According to mean global hospitalization cost, no difference was found (22,020 euros +/− 10,082 vs 17,976 euros +/− 14,434, *p* = 0.43).

## Discussion

Although recent advances in surgical technique and perioperative care have resulted in a lower mortality rate in patients who undergo pancreatic resection, the morbidity rate remains high, ranging from 20% to 60%, including 20% to 30% with Clavien-Dindo grade 3 to 4 complications [40–44]. Here, we study the efficacy on DGE and postoperative complications of NG, GT and GJ tube with different types of nutrition (TPN, EN) after PD. The mortality rate was 6% and was in accordance with the literature [3–6], with no difference between the three groups. However, the morbidity rate was 73%, including 55% severe morbidity (Clavien-Dindo grade 3/4), and tended to be higher in the GT and GJ groups in univariate analyses. These results could be explained by the fact that patients in these groups were smoker and had an ASA score of 3, more often than in the NG group and presented with more postoperative thromboembolic complications and respiratory and cardiac failures that required intensive care unit hospitalization.

Among PSCs, DGE represents the most frequent complication after PD (15–60%) [14–18]. Several gastric decompression systems exist to manage this complication. DGE acts to significantly increase the length of hospital stay and cost and also impairs quality of life [19–22]. Recently, the concepts of “fast-track” surgery, which provides optimal perioperative care, have been proven to reduce complication rates and decrease the length of hospital stay [11–13]. However, the quality of evidence on the application of fast-track clinical protocols in pancreatic surgery is moderate, and they have not gained widespread acceptance. Furthermore, when DGE occurs, oral feeding is stopped and a nasogastric tube is inserted [45–47].

In the present study, we were able to show that a GT tube with TPN is an independent risk factor for postoperative DGE (B/C) compared to NG and GJ tubes. Many hypotheses to explain DGE have been studied: (a) some authors have reported that the presence of intra-abdominal complications is the most important risk factor for DGE [45, 48]. However, no difference was found between the three groups concerning the intra-abdominal complications rate in our series. (b) All patients in the GT group and GJ group had pylorus conservation. However, no difference was found between the two groups concerning the DGE rate. The impact of pylorus conservation remains under debate. Some studies have shown no difference in the incidence of DGE irrespective of whether the pylorus is preserved [49, 50] while in others [51, 52] pylorus-preserving PD is an independent predictive factor for DGE. Current evidence suggests that given obvious clinical (DGE definition) and methodological heterogeneity, future high-quality randomized controlled trials of complex surgical interventions based on well-defined outcome factors are required [52, 53]. (c) All patients with a GT tube in our series had TPN; however, conflicting results have been reported concerning the DGE rate found between oral diet, EN, and TPN after PD [33, 34]. We assume that the cause of this difference in postoperative gastroparesis rates between different groups is multifactorial. First, more than the presence of an intra-abdominal complication, the nature, size, and position of the intra-abdominal complication may be responsible for postoperative DGE. Second, the disadvantages caused by gastric decompression devices are not the same. The GJ tube and GT tube are better accepted by patients than the NG tube, NG tube insertion has been associated with higher inconvenience for patients. In addition, it may cause patient discomfort and is associated with nasal trauma, gastroenteral reflux, respiratory complications, and can lead to the dislodgement of the NG tube [23–26]. As a result, the GJ tube and GT tube are kept in place longer, because the surgical teams are still reticent to apply tension to the gastrojejunal anastomosis and fear the consequences of a clinically relevant POPF. The DGE rate and the DGE grade in the GJ and GT groups could therefore be overestimated. Mack et al. reported that EN via a GJ tube insertion was associated with a reduced incidence of DGE compared to an NG tube with TPN [27]. In contrast we showed no difference between a GJ tube with EN and an NG tube with TPN concerning postoperative DGE rates, but the gastric decompression mean duration was lower in the NG group than in the other two groups.

The most clinically relevant surgical complication after PD is the POPF that occurred in approximately 20%–30% of patients [54–56]. POPF is a major source of various other complications such as sepsis, intra-abdominal abscesses, DGE, hemorrhage, prolongation of the hospital stay, and the cost of the treatment. Several risk factors have been reported, such as higher body mass index, soft pancreatic texture, main pancreatic duct size ≤3 mm, large pancreatic remnant, additional procedures, and sarcopenia [56–62]. In our series, patients with a GJ tube associated with EN and a GT tube with TPN had a higher clinically relevant grade C POPF than patients with an NG tube with TPN in univariate analysis. However, as reported by Mack et al. [27], the multivariate analysis did not highlight either gastric decompression devices or feeding protocols as independent risk factors of POPF. Our results could be explained by the sample size in the group with a GJ tube with EN.

The choice between enteral, parenteral nutrition or a combined enteral-parenteral nutritional approach after PD remains under debate [28–30, 33, 34, 63]. Recently, a multicenter randomized controlled trial compared nasojejunal early EN to TPN after PD in terms of postoperative complications [64] and showed that nasojejunal early EN was associated with an increased overall postoperative complication rate. Furthermore, the frequency and the severity of POPF were also significantly increased after using this feeding system. Thus, authors did not recommend nasojejunal early EN [64]. Two recent reviews and meta-analyses, which included randomized controlled trials, meta-analyses or well-designed studies, determined the optimal feeding route and the correlation between EN, main complications, and outcome after PD [28, 34]. EN appears to be safe and tolerated in patients but does not have clear advantages in reducing postoperative DGE, POPF, postoperative hemorrhage, infectious complications, and length of hospital stay [28, 34]. As shown in our study, we reported that a GJ tube with EN has no clear advantage in reducing PSCs when compared to other systems.

PSCs, notably including DGE, can prolong the hospital length of stay. Mack et al. found that insertion of a GJ tube and enteral feeding were associated with a shorter length of hospital stay and cost savings. However, cost analysis in this study used assumptions and modeling to extrapolate cost predictions [27]. Here, we showed that a GT tube with TPN and a GJ tube with EN are two independent risks factors for greater postoperative length of hospital stay as compared to an NG tube, probably because devices caused less discomfort than an NG tube and were removed later. As reported in the literature [5, 19–22], we found that PSCs and preoperative jaundice increase significantly the length of hospital stay. However, multivariate analysis did not highlight the GJ tube, GT tube, or feeding strategy as independent risk factors of PSCs. Furthermore, no difference was found concerning the rate of preoperative jaundice between the three groups.

Considering the costs of different devices, nutritional protocols, and length of hospital stay, we found no advantages to EN through a GJ tube when compared to an NG tube or a GT tube with TPN in our series. Although TPN is more expensive, the length of hospital stay in the group of patient with EN, largely offsets the price differential between TPN and EN. Mean nutrition cost and mean global hospitalization cost were not decreased in the GJ group when compared to the NG group, and GT tube insertion with TPN nutritional protocol appeared as the most expensive for nutrition and hospitalization length of stay. However, the small number of patients in each group probably explains the low power of statistical analysis and the lack of significant difference. Further analyses on larger and prospective series are warranted for confirming the robustness of these results.

Our study has several biases. First, data were obtained within a multicenter study, and reconstruction was undertaken according to the surgeon’s preference. Second, this study is limited by the non-random allocation of patients to the different feeding tube techniques. According to the center preference, three different gastric decompression and feeding systems were used. Third, the sample size in the enteral nutrition group is small.

## Conclusions

In conclusion, gastrostomy tube insertion with TPN was associated with increased severe postoperative morbidity and DGE and should not be recommended. EN through a double-lumen gastrojejunostomy tube after PD is feasible but does not have clear advantages in reducing mortality, morbidity, hospital length of stay, and cost savings compared to an NG tube.

## Abbreviations

ASA: American society of anesthesiologists
CI: Confidence interval
DGE: Delayed gastric empty
DRG: Diagnosis-related group
EN: Enteral nutrition
GJ: Gastrojejunostomy
GLM: Generalized linear model
GT: Gastrostomy
ISGPS: International study group of pancreatic surgery
OR: Odd ratio
PD: Pancreaticoduodenectomy
POD: Postoperative day
POPF: Postoperative pancreatic fistula
PPPD: Pylorus preserving pancreaticoduodenectomy
PSC: Pancreatic specific complication
PVR: Portal vein resection
SD: Standard deviation
TPN: Total parenteral nutrition
